# Author Correction: Glacigenic sedimentation pulses triggered post-glacial gas hydrate dissociation

**DOI:** 10.1038/s41467-018-03587-0

**Published:** 2018-03-07

**Authors:** Jens Karstens, Haflidi Haflidason, Lukas W. M. Becker, Christian Berndt, Lars Rüpke, Sverre Planke, Volker Liebetrau, Mark Schmidt, Jürgen Mienert

**Affiliations:** 1 Department of Earth Science, University of Bergen, Bergen, Norway; 2 GEOMAR Helmholtz Centre for Ocean Research Kiel, Kiel, Germany; 3 Volcanic Basin Petroleum Research (VBPR), Oslo, Norway; 4 The Centre for Earth Evolution and Dynamics (CEED), University of Oslo, Oslo, Norway; 5 CAGE—Centre for Arctic Gas Hydrate, Environment and Climate, UiT—The Arctic University of Norway, Tromsø, Norway

Correction to: *Nature Communications* 10.1038/s41467-018-03043-z, published online 12 February 2018

The original version of this Article contained an error in the second sentence of the Abstract, which incorrectly read ‘They are stable under high pressure and low, but react sensitively to environmental changes.’ The correct version adds ‘temperature’ after ‘low’. This has been corrected in both the PDF and HTML versions of the Article.

